# Drug Repurposing to Enhance Antitumor Response to PD-1/PD-L1 Immune Checkpoint Inhibitors

**DOI:** 10.3390/cancers14143368

**Published:** 2022-07-11

**Authors:** Xavier Thuru, Romain Magnez, Hassiba El-Bouazzati, Gérard Vergoten, Bruno Quesnel, Christian Bailly

**Affiliations:** 1 University of Lille, CNRS, Inserm, CHU Lille, UMR9020-UMR1277—Canther—Cancer Heterogeneity Plasticity and Resistance to Therapies, F-59000 Lille, France; xavier.thuru@univ-lille.fr (X.T.); romain.magnez@inserm.fr (R.M.); hassiba.elbouazzati@inserm.fr (H.E.-B.); bruno.quesnel@chru-lille.fr (B.Q.); 2 Institut de Chimie Pharmaceutique Albert Lespagnol (ICPAL), Faculté de Pharmacie, University of Lille, Inserm, INFINITE—U1286, 3 Rue du Professeur Laguesse, BP-83, F-59006 Lille, France; gvergoten@orange.fr; 3 Oncowitan, Scientific Consulting Office, F-59290 Lille, France

**Keywords:** azelnidipine, cancer therapy, drug repurposing, immune checkpoint, liothyronine, niclosamide, PD-1, PD-L1, tumor

## Abstract

**Simple Summary:**

Novel pharmacological approaches are needed to improve treatments of advanced cancers, despite the considerable benefit of immunotherapy. New drugs are searched for to complement the activity of monoclonal antibodies targeted to the PD-1/PD-L1 immune checkpoint. Here, we have identified and discussed known drugs which could reinforce immunotherapy based on their capacity to modulate this major checkpoint. The repositioning of these drugs, in particular liothyronine, azelnidipine, niclosamide, and albendazole, may represent an alternative approach to improve cancer treatments, compared to the de novo drug design strategy. The repurposing of a few other established drugs to promote cancer immunotherapy is also presented.

**Abstract:**

Monoclonal antibodies targeting the PD-1/PD-L1 immune checkpoint have considerably improved the treatment of some cancers, but novel drugs, new combinations, and treatment modalities are needed to reinvigorate immunosurveillance in immune-refractory tumors. An option to elicit antitumor immunity against cancer consists of using approved and marketed drugs known for their capacity to modulate the expression and functioning of the PD-1/PD-L1 checkpoint. Here, we have reviewed several types of drugs known to alter the checkpoint, either directly via the blockade of PD-L1 or indirectly via an action on upstream effectors (such as STAT3) to suppress PD-L1 transcription or to induce its proteasomal degradation. Specifically, the repositioning of the approved drugs liothyronine, azelnidipine (and related dihydropyridine calcium channel blockers), niclosamide, albendazole/flubendazole, and a few other modulators of the PD-1/PD-L1 checkpoint (repaglinide, pimozide, fenofibrate, lonazolac, propranolol) is presented. Their capacity to bind to PD-L1 or to repress its expression and function offer novel perspectives for combination with PD-1 targeted biotherapeutics. These known and affordable drugs could be useful to improve the therapy of cancer.

## 1. Introduction

Monoclonal antibodies (mAbs) targeting the programmed cell death 1 (PD-1) receptor or its ligand PD-L1 are increasingly used for the treatment of multiple forms of cancer, especially solid tumors. There are currently 13 mAbs registered, including 10 directed against PD-1 and 3 targeting PD-L1 [1]. The first anti-PD-1 mAbs, nivolumab and pembrolizumab, were approved by the US Food and Drug Administration (FDA) in 2014 and the first anti-PD-L1 mAb, atezolizumab, received FDA approval in 2016. The family continues to expand with new mAbs regularly approved by different health authorities. The most recent anti-PD-1 mAb, dostarlimab, was approved in 2021 for the treatment of advanced endometrial cancer [2]. In addition, there are multiple mono- or bispecific antibodies (and fragments or fusion proteins) targeting PD(L)-1 currently in clinical development. To cite only one example, the (LAG-3 × PD-L1) bispecific mAb ABL501 has recently entered phase 1 trial in patients with locally advanced (unresectable) or metastatic solid tumors (NCT05101109) [3]. Many other bispecific antibodies (BsAbs) targeting the PD(L)-1 checkpoint and another immune checkpoint or cancer target are undergoing development [1,4,5].

There is no doubt that the use of anti-PD-(L)1 mAbs has profoundly changed the therapies of cancers and significantly improved patient survival. However, the efficacy of immune checkpoint inhibitors (ICIs) varies considerably from one tumor type to another and among different patient populations. For example, patients with advanced non-small-cell lung cancer (NSCLC) or a melanoma expressing high PD-L1 levels, in general, respond very well to pembrolizumab or atezolizumab. Good responses have been observed also for patients with head and neck squamous cell carcinoma and urothelial carcinoma [6,7,8]. However, for other cancers, the antitumor activity in patients is much less impressive and alternative strategies are needed. In fact, immune checkpoint blockade therapies induce durable tumor regressions in a minority of patients with cancer. For example, the response rate to anti-PD-1 monotherapy rarely exceeds 20–30% in patients with advanced-stage hepatocellular carcinoma [9,10,11]. In other cases, the anti-PD-(L)1 treatment is efficient, but secondary resistance occurs in most patients. This is the case for patients with head and neck squamous cell carcinoma (HNSCC), for example [12]. Similarly, in oncohematology these ICIs have been used with limited success for the treatment of patients with relapsed or refractory acute myeloid leukemia (AML) [13]. PD-1 and PD-L1 both contribute to maintaining a strongly immunosuppressive tumor microenvironment, which favors the clonal evolution of blasts [14]. Insufficient clinical responses have been noted in patients with AML or myelodysplastic syndromes (MDSs) upon treatment with these ICIs [14].

For these reasons, novel treatments are needed to combat resistant tumors and to offer a better perspective to patients with advanced cancers. Several options have been proposed, in particular, combinatorial therapies which consist of a cumulation of different treatment modalities, such as immunotherapy combined with chemotherapy [15], anti-angiogenic therapy [16], radiotherapy [17,18], and other treatment options (targeted therapeutics, nanotechnologies, etc.). There are many possibilities of combination therapies to improve the anticancer efficacy of a PD-1/PD-L1 blockade [19]. Just to cite one example, promising data were obtained when combining the multikinase inhibitor regorafenib with ICIs. The small molecule induced PD-L1 degradation and promoted the efficacy of an anti-PD-1 mAb to treat advanced colon cancer [20,21,22].

**Figure 1:**
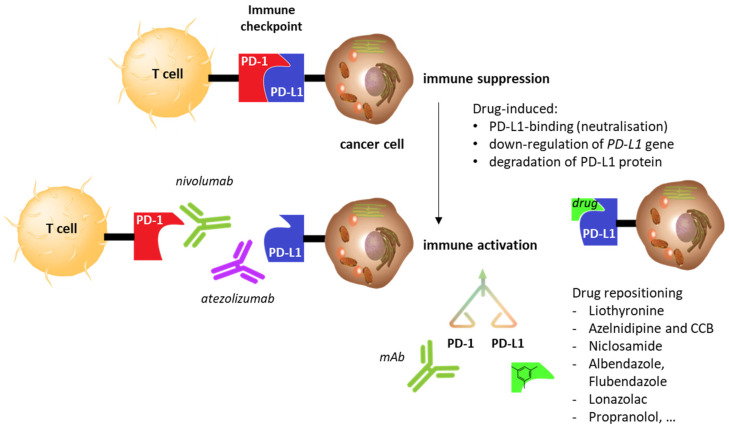
Schematic of the PD-1/PD-L1 immune checkpoint and its blockade with monoclonal antibodies (mAbs). There are currently 13 registered antibodies, 10 against PD-1 (nivolumab, avelumab, cemiplimab, pembrolizumab, toripalimab, sintilimab, camrelizumab, tislelizumab, zimberelimab, prolgolimab, dostarlimab) and 3 against PD-L1 (atezolizumab, durvalumab, avelumab). Anti-PD-(L)1 mAbs are used to reactivate the immune system. A comparable effect can be obtained with small molecules directed against PD-L1, either binding/blocking the ligand or inducing its down-regulation or degradation via indirect effectors. Repositioned drugs that impact PD-L1 activities are discussed here.

An alternative approach also tested consists of combining the ICI with other drugs not primarily used to treat cancer, but that still have an effect on the checkpoint. The drug of interest could either modulate the expression of the immune checkpoint (receptor or ligand), or induce a degradation of the PD-L1 ligand expressed on cancer cells, or contribute to strengthening the blockade of the PD-1/PD-L1 interaction via binding to PD-L1 (Figure 1).

These approaches of discovering different uses for existing drugs are referred to as repositioning, reprofiling, or repurposing strategies. Drug repurposing is generally considered to reposition old, clinically approved off-patent noncancer drugs with known targets into newer indications [23]. It is an attractive process, susceptible to bringing new therapeutics at a much lower cost compared to the expensive and long development of a new chemical entity [24,25]. The repurposing of small molecules is also intensely considered at present, with about 100 non-oncology small-molecule drugs in clinical development worldwide [26]. For example, the repositioning of anti-parasitic and anti-bacterial drugs has been largely considered for the treatment of cancer, with promising perspectives to combat difficult-to-treat tumors such as pancreatic cancer [27,28,29]. In this context, we have identified several drugs which could be used to modulate the PD-1/PD-L1 checkpoint so as to offer a synergistic action with a mAb targeting PD-1 or PD-L1. The different cases are discussed in turn in the following subsections.

## 2. Drug Repositioning to Target the PD-1/PD-L1 Checkpoint 

### 2.1. Repositioning of Liothyronine as a PD-L1 Binding Agent

The thyroid gland synthesizes different hormones including triiodothyronine (T3) and its precursor, thyroxine (T4), which are important in the regulation of body homeostasis. T4 is secreted by the thyroid gland in response to the thyroid-stimulating hormone (TSH) originating from the pituitary gland. Most of the conversion of T4 to T3 occurs outside the thyroid. T3 is the metabolite of the prohormone thyroxine. The natural hormone *levo*-triiodothyronine (L-T3) is essential for DNA transcription, mitochondrial biogenesis, and respiration. The maintenance of a correct serum level of this hormone is important, as low levels of T3 in cardiac patients are associated with worse outcomes. On the opposite hand, short-term T3 therapy is considered in patients undergoing cardiac surgery or those with cardiovascular diseases [30]. There is a key crosstalk between the endocrine system and the immune system, with important modulation of the activity of T cells in the presence of T3. In general, a hyperthyroid state leads to a more activated immune system, whereas hypothyroidism leads to a less activated immune system [31]. The crosstalk explains the cases of thyroid immune-related adverse events which are not uncommon in patients treated with a PD-1 mAb. Destructive hypothyroidism, generally reversible, is relatively common in cancer patients treated with pembrolizumab or nivolumab [32,33,34].

Recent studies have demonstrated that T3 controls T cell activity via dendritic cell (DC) modulation, and specifically via a proinflammatory response mediated by interleukin-17 (IL-17). T3 has the capacity to down-modulate PD-1 expression on CD4^−^ cells, as to limit the immune inhibitory signal driven by this co-inhibitory pathway [35]. In addition, T3 can modulate the secretion of angiogenic growth factors and cytokines in specific situations [36]. The idea of using thyroid hormones to modulate activities of immune cells has been studied for many years now [37,38], but recent data have specifically demonstrated the link between T3 and the PD-1/PD-L1 checkpoint [35].

Liothyronine is the synthetic L-form of triiodothyronine (L-T3, Cytomel^®^) and levothyroxine is a synthetic L-form of tetraiodothyronine (L-T4). Liothyronine is used to treat congenital or acquired hypothyroidism and as an adjunct therapy to surgery and radioiodine in the management of thyroid cancer. It is a convenient oral product used to alleviate thyroid dysfunctions by replacing insufficient hormonal production and restoring T3 plasma levels. Upon binding to the thyroid hormone receptor β (TRβ), the compound can increase the viability of dendritic cells, stimulate their migration to lymph nodes, and potentiate their immunogenicity. By doing so, T3 enhances the ability of dendritic cells to stimulate a cytotoxic T cell response. In other words, the drug behaves as a DC instructor to stimulate a T cell-mediated antitumor response [39,40]. It drives a proinflammatory response via the production of interleukin-17 [35]. 

**Figure 2:**
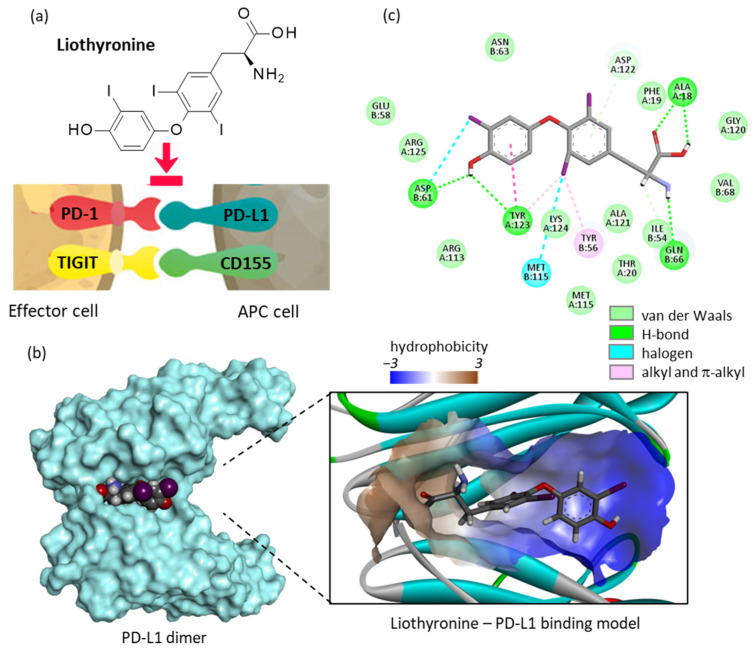
Liothyronine binding to PD-L1. (**a**) Structure of liothyronine (L-T3). The compound has been shown to interfere with the two immune checkpoints PD-1/PD-L1 and TIGIT/CD155. (**b**) A molecular model of liothyronine bound to a dimer of PD-L1, with a close-up view of the binding site at the interface of the two monomers and the hydrophobic/hydrophilic areas (color code indicated). (**c**) Binding map contacts for liothyronine bound to PD-L1. The empirical energy of interaction (ΔE= −67.22 kcal/mol) and energy of hydration (ΔG = −14.10 kcal/mol) were calculated. The docking analysis was performed as previously described [43].

Liothyronine has been identified recently as a potential PD-L1-binding drug in the frame of a virtual (in silico) drug screening procedure. The authors suggested that the compound could form stable complexes with PD-L1, notably via the π-π stacking of the central phenyl ring of T3 with the tyrosine 123 residue of PD-L1. Additional H-bond and hydrophobic interactions also contribute to the stability of the T3/PD-L1 complex [41] (Figure 2). Tyrosine Y123 is known as a critical residue for both PD-L1 dimerization and PD-1/PD-L1 binding [42]. Based on this observation, it has been proposed to use liothyronine not only to reduce the risk of hypothyroidism, but also to further inhibit the PD1/PD-L1 interaction and to reduce expression of the T3-precursor (T4). It would be a clever option to boost the immune system to fight against cancer cells [41]. However, at present, this computational prediction has not received an experimental validation to our knowledge.

The frequent occurrence of immune-related adverse events (irAEs) of the thyroid caused upon inhibition of the PD-1/PD-L1 checkpoint can raised questions and fears regarding the use of a drug acting on the thyroid. Thyroid dysfunction is the most common endocrine irAE in patients treated with anti-PD-1 mAbs (at least 20% of patients). Severe hypothyroidism is a dangerous situation, possibly leading to dilated cardiomyopathy and decreased heart function. Anti-PD-1 antibodies can cause thyrotoxicosis and hypothyroidism [44]. Liothyronine, being classically used to treat hypothyroidism, could be useful to reduce the risk of irAEs of the thyroid caused by anti-PD-1 mAbs. Nevertheless, the potential use of liothyronine in combination with an ICI would require careful and regular monitoring of thyroid functions, as is already the case for different immune checkpoint inhibitor therapies [45].

Liothyronine stands as a very interesting immunoactive compound for another reason. It has the capacity to bind to the cell surface of glycoprotein CD155 (also known as the poliovirus receptor PVR) which is a checkpoint ligand for TIGIT (T cell immunoreceptor with immunoglobulin and immunoreceptor tyrosine-based inhibitory motif domains). Like PD-1, TIGIT is a major inhibitory immune checkpoint molecule [46,47]. The blockade of TIGIT enhances the NK cell response and the antitumor effector T cell response, and reduces the suppressive capacity of regulatory T cells [48]. In the frame of a virtual screening, liothyronine has been identified as a potential ligand for CD155, binding to the site proximal to the TIGIT/CD155 interaction zone. In this case, the prediction has been validated experimentally. Liothyronine was found to block the interaction of CD155 with TIGIT in a dose-dependent manner and an IC_50_ value of 6.1 μM was calculated [49]. In parallel, the capacity of liothyronine to bind to CD155 was evaluated by means of microscale thermophoresis (MST) and K_D_ values of 2.64 and 0.36 μM were determined using murine and human CD155, respectively. The binding of liothyronine to a binding pocket of CD155 prevents the protein from interacting with TIGIT. In this experimental study, the authors indicated that liothyronine functions as a blocker of the TIGIT/CD155 checkpoint, but was unable to block the PD-1/PD-L1 checkpoint [49]. This is in contradiction with the (more recent) in silico study mentioned above [41]. Unfortunately, the predictability of computational studies is often limited, despite the development of more and more sophisticated methods.

It is remarkable that liothyronine can function as an immune checkpoint blocker, at least for the TIGIT/CD155 checkpoint, and this effect enhances significantly the anticancer action of T cells. It was clearly shown that the drug could suppress tumor growth and stimulate CD8^+^ T cell response in mice bearing a syngeneic MC38 colon tumor. It is apparently a remarkable drug to enhance tumor infiltration by CD8^+^ T cells [41]. Whether liothyronine is able to modulate the PD-1/PD-L1 checkpoint, in addition to the TIGIT/CD155 checkpoint, remains to be clarified. Nevertheless, it is an interesting drug for a drug repositioning strategy to treat cancer. To our knowledge, no clinical trial with liothyronine for the treatment of cancer has been initiated yet. It is time now to design appropriate trials with this drug.

### 2.2. Repositioning of Dihydropyridine Calcium Channel Blockers to Treat Cancer

**Figure 3:**
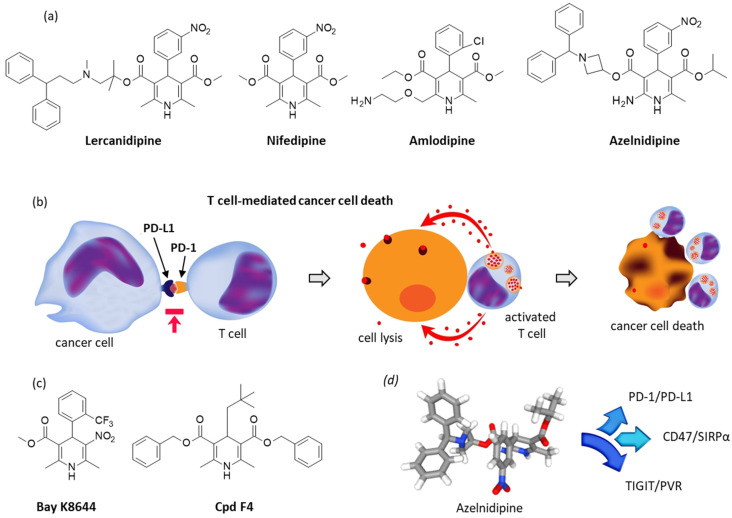
Dihydropyridine-type calcium channel blockers (CCBs) and their PD-1/PD-L1-mediated activity. (**a**) Structure of four typical CCBs. (**b**) Schematic action of their PD-L1-mediated action. (**c**) Structures of the calcium channel agonist BayK8644 and the PD-L1 degrader F4. (**d**) Molecular model of azelnidipine and its activity toward immune checkpoints.

Dihydropyridine-type calcium channel blockers (CCBs) represent a group of potent vasodilators used for the treatment of hypertension and chronic coronary artery disease. They are also used to combat chronic kidney disease and diabetic nephropathy in some cases [50]. The family comprises about 20 compounds, including amlodipine, nifedipine, azelnidipine, lercanidipine (Figure 3a), and several others [51]. New calcium channel blocking compounds with a selective action on subtypes of the L-channel continue to be developed, notably to reduce the risk of drug dependence [52].

Beyond cardiovascular diseases, these drugs have revealed an interesting use for the treatment of cancers. Recently, the CCB amlodipine (commonly used to treat arterial hypertension) was found to markedly enhance the therapeutic response to gemcitabine chemotherapy in pancreatic cancer, extending survival and reducing the risk of distant metastases [53]. In addition, the drug was found to enhance the response to the multikinase inhibitor regorafenib in patients with metastatic colorectal cancer [54]. Several in vitro/in vivo studies have revealed that amlodipine, lercanidipine, and other structurally related CCBs can exert an antiproliferative action toward cancer cells and/or promote the efficacy of different anticancer drugs [55,56,57,58,59,60]. For example, the L-type CCB manidipine provided a synergistic combination with the pan-HER kinase inhibitor poziotinib to induce apoptosis in ovarian cancer stem cells [61,62]. Based on all these different observations, the repositioning of a CCB such as amlodipine for cancer therapy has been proposed [63].

The idea was resurrected recently with the observations that CCBs can inhibit activation of the transcription factor STAT1, thereby suppressing the transcription of the *PD-L1* gene. This has been demonstrated with the CCB lercanidipine that is capable of down-regulating PD-L1 in lung cancer cells (NCI-H1299 cells and NCI-H460 cells) and enhancing the killing ability of T cells. A similar capacity to induce T cell-mediated cancer cell death was then evidenced with azelnidipine and amlodipine, although these two other CCBs were slightly less potent than lercanidipine [64] (Figure 3b). In another similar study, amlodipine was found to induce of PD-L1 degradation and antitumor immunity in a mouse MC38 tumor model. The drug selectively induced the autophagic degradation of PD-L1 in a calcium-dependent manner [65]. These two independent studies point to the interest of dihydropyridine-type CCBs to modulate expression of PD-L1 in tumor cells. Moreover, the related drug nifedipine was shown previously to decrease PD-L1 expression on colorectal cancer cells and to reactivate tumor immune monitoring by T cells. The effect was indirect. It is the inhibition of calcium influx by nifedipine which alters the dephosphorylation, activation, and nuclear translocation of the transcription factor NFAT2 (nuclear factor of activated T cell 2) and, subsequently, prevents proliferation and metastasis of the colorectal cancer cells [66].

At this point, it is useful also to evoke the calcium channel agonist BayK8644 (Figure 3c) which has been recently characterized as a potent inhibitor of the transmembrane protein 176B (TMEM176B, also known as TORID for tolerance-related and induced) [67]. This protein is an endophagosomal immunoregulatory cation channel functioning as an inhibitor of activation of the NLRP3 inflammasome through the control of cytosolic Ca^2+^. Inhibition of TMEM176B by the 1,4-dihydropyridine derivative BayK8644 triggers inflammasome-dependent tumor control and improves the efficacy of immune checkpoint blockers, such as anti-CTLA4 and anti-PD-1 monoclonal antibodies. BayK8644 was found to enhance significantly the antitumoral effect of anti-PD-1 therapy in mice bearing a melanoma tumor through the potentiation of CD8^+^ T cell-dependent antitumor immunity [67]. However, the exact mode of action of this Ca channel activator is unclear. Recently, this compound was shown to promote the growth of human liver cancer HepG2 cells in vitro [68]. The activity of the compound is apparently solvent-dependent. A study performed 30 years ago indicated that in DMSO, BayK8644 is a T channel antagonist, but an L-channel agonist in an ethanol:water mixture [69]. Dihydropyridine-type calcium channel antagonists (drugs), and also this specific agonist BayK8644 (a laboratory tool), can be used to modulate the PD-1/PD-L1 checkpoint.

Dihydropyridine CCBs warrant further studies as potential modulators of the PD-1/PD-L1 checkpoint. As mentioned above, studies have been performed with lercanidipine, amlodipine, and a few other similar compounds, such as azelnidipine, although this later compound is less potent than lercanidipine at down-regulating PD-L1 and inducing T cell-mediated cancer cell death [64]. Nevertheless, azelnidipine is a compound of prime interest for another reason: it is an inhibitor of two other immune checkpoints CD47/SIRPα and TIGIT/PVR. The drug has been found to bind to the isolated proteins hSIRPα (K_D_ = 5.4 μM) and hPVR (K_D_ = 6.5 μM) using microscale thermophoresis. In both cases, a potential binding pocket was identified and the drug was found to enhance phagocytosis of tumor cells by macrophages. In vivo, azelnidipine only slightly reduced the growth of a CT26 colon tumor in mice, but a much more pronounced effect was observed upon combination with a local radiation of the tumor. The proportion of CD8^+^ T cells producing interferon-γ was enhanced upon treatment with azelnidipine (5 mg/kg) in tumor-bearing mice [70]. This CCB appears as an interesting anticancer agent, well suited for a repositioning strategy (Figure 3d). Its mechanism of action is probably multifactorial, implicating different immune checkpoints such as PD-1/PD-L1, CD47/SIRPα, and TIGIT/PVR, and possibly other targets. Very recently, the anticancer effect of azelnidipine was evidenced in a mouse xenograft model of liver cancer and associated with the down-regulation of the enzyme tryptophan 2,3-dioxygenase [71]. However, no clinical trial for the treatment of cancer with a CCB has been reported at present.

The demonstration that lercanidipine can trigger PD-L1 degradation in cancer cells [62] has encouraged the design of newer dihydropyridine derivatives with a reduced calcium influx antagonistic activity, but that retain a PD-L1 degradation activity. The compound F4 (Figure 3c) has been identified as a PD-L1 degrader capable of strengthening the T cell-mediated killing of tumor cells, possibly via a lysosomal mechanism [72]. Dihydropyridine CCBs have not finished revealing their anticancer potential. They can be used to modulate immune response against tumor cells.

### 2.3. Repositioning of Niclosamide as a STAT3-Dependent Regulator of the PD-1/PD-L1 Checkpoint

Niclosamide (NCS) has been used to treat tapeworm infection in humans for decades. This old FDA-approved anthelmintic drug, recommended by the World Health Organization (but not available in the US), is used to treat parasitic infections in millions of people worldwide [73]. Beyond its molluscicidal effect, niclosamide has revealed a myriad of other pharmacological effects of interest, notably for the treatment of cancers and virus infections [74]. In addition, over the past three years, in the frame of the SARS-CoV-2 pandemic crisis, the potential repositioning of niclosamide to treat COVID-19 disease has been largely investigated. The drug presents marked antiviral and anti-inflammatory activities, as well as a bronchodilatory effect potentially useful to treat COVID-19 patients [75]. Clinical trials are still ongoing, but in a phase 2 study recently published, niclosamide did not reveal the expected effect on the duration of symptoms in COVID-19 patients [76]. Other trials are in progress and a clinical benefit has been reported [77].

The potential repurposing of NCS for the treatment of cancers has been extensively described. There are many studies evidencing the capacity of the compound to reduce tumor growth in diverse models of tumor-bearing mice. Clinical trials using NCS have also been deployed, notably for the treatment of metastatic colorectal cancer [78,79]. The anticancer mechanism of action of NCS is complex and multifactorial. Several molecular targets and pathways have been implicated, including degradation of β-catenin induced upon phosphorylation of glycogen synthase kinase-3 (GSK-3β) [80,81,82]. This small molecule can be combined with conventional cytotoxic agents such as camptothecin or temozolomide to treat glioblastoma [83,84], with paclitaxel or doxorubicin to treat triple-negative breast cancer [85,86], and with other drugs used to treat colon cancer, prostate cancer, osteosarcoma, etc. [87,88]. NCS functions also as a STAT3 inhibitor, useful to enhance the efficacy of diverse types of cytotoxic drugs and targeted therapeutics [89,90,91].

**Figure 4:**
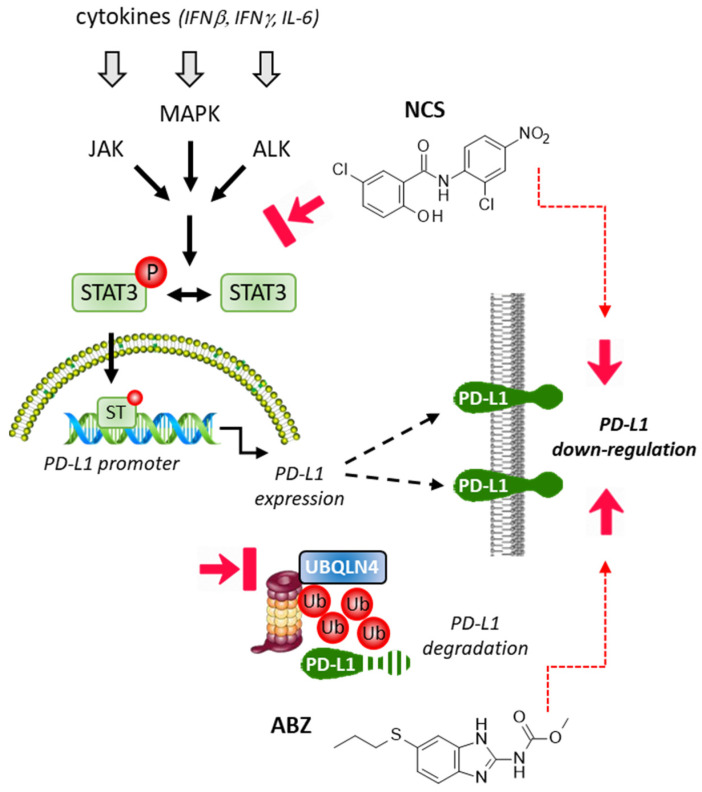
Inhibition of STAT3 activation by niclosamide (NCS) leading to a decrease in *PD-L1* gene expression and a down-regulation of PD-L1 protein expressed at the surface of cancer cells. Reduced expression of ubiquilin 4 (UBQLN4) induced by albendazole (ABZ) triggers ubiquitination of PD-L1 and its subsequent ubiquitination by the proteasome. This effect leads to a down-regulation of membrane-bound PD-L1.

An interesting work has described the capacity of NCS to promote the anticancer activity of an anti-PD-L1 antibody in an experimental model of NSCLC. NCS was found to enhance the lysis of the cancer cells by T cells, increasing the infiltration of the tumor by those T cells and the release of cytolytic granzyme B. The effect was coupled with a concentration- and time-dependent decrease in the expression of PD-L1 on the cancer cells in the presence of NCS (Figure 4). It is apparently the blockade of the binding of phospho-STAT3 to the *PD-L1* promoter which is at the origin of the antitumor effect [92]. A down-regulation of *PD-L1* induced by NCS has been reported in another recent study with a model of pancreatic cancer and, in this case, the immune effect of NCS promoted the anticancer activity of the drug gemcitabine [82].

The specific combination of NCS with an anti-PD-1 mAb points to a more general effect, which is the inhibition of the PD-1/PD-L1 checkpoint signaling via activation of the STAT3 pathway. There are multiple examples of drugs, chemicals, and natural products capable of promoting PD-1 or decreasing PD-L1 expression via a STAT3-dependent action [93,94,95,96,97]. STAT3 is a master regulator of the PD-1/PD-L1 immune checkpoint [98]. In brief, NCS is a good candidate for repurposing in oncology, but the active principle should probably be reformulated because it has a poor aqueous solubility and a low bioavailability. The use of cyclodextrin–NCS complexes, polymeric micelles, or specific nanoparticles containing NCS have been proposed to improve the anticancer efficacy of the compound [85,86,99,100].

Multiple clinical trials with NCS for the treatment of cancers have been performed or initiated recently. The drug is being tested for the treatment of acute myeloid leukemia (NCT05188170), colon cancer (NCT02687009, NCT04296851, NCT02519582) and hormone-resistant prostate cancer (NCT03123978, NCT02532114, NCT02807805). However, no clinical trial of NSC combined with an anti-PD-(L)1 mAb has been reported.

### 2.4. Albendazole and Flubendazole to Modulate the PD-1/PD-L1 Checkpoint

For a long time, benzimidazole-based drugs were used to treat infectious diseases in humans and animals caused by parasitic helminths (worms such as *Ascaris lumbricoides*, *Ancylostoma duodenale*, and *Trichuris trichiura*). Helminth parasites cause significant morbidity and mortality in endemic countries. More than a quarter of the world’s population (approximately 2 billion people) are affected by helminthic parasites [101]. Benzimidazole derivatives are certainly the most widely used compounds to combat these parasites. The family of compounds include well-known drugs such as albendazole (ABZ) and mebendazole, but also several other representatives such as fenbendazole, oxfendazole, thiabendazole, triclabendazole, parbendazole, ricobendazole, and oxibendazole. Some of these drugs have been used for a very long time, such as thiabendazole (year of US approval: 1967), mebendazole (1974), and albendazole (1996), but there are also recent derivatives, such as triclabendazole (Egaten^®^, Basel, Switzerland), which was approved in 2019 for the treatment of fascioliasis (a parasitic worm infection caused by the common liver flukes *Fasciola hepatica* and *F. gigantica*) [102,103]. Mebendazole is being tested clinically for the treatment of different forms of cancers, such as colon cancer (NCT03925662, NCT03628079), liver cancer (NCT04443049), and brain tumors (NCT01729260, NCT02644291, NCT01837862). However, no clinical trial in association with an anti-PD-(L)1 mAb has been declared.

In addition to their primary antiparasitic effects, most of these benzimidazole derivatives have revealed interesting anticancer properties, which has encouraged the design of benzimidazole-containing anticancer drugs [104] and the repositioning of these antiparasitic drugs for the treatment of cancer. The anticancer properties of drugs such as albendazole and fenbendazole have been amply reported [26,105,106]. The most potent compound in the series is certainly flubendazole (FLU) [107,108]. We only focus on the capacity of this compound to modulate the PD-1/PD-L1 checkpoint.

FLU exhibits remarkable anticancer effects. The drug has shown efficacy in models of breast, lung, and skin cancers, and cancer of the oral cavity. Its mechanism of action is multifactorial, including cell cycle effects, a decrease in cancer cell stemness, suppression of cancer cell proliferation and induction of apoptosis, inhibition of cell migration, modulation of drug resistance, and, importantly here, silencing of the immune suppressive effects of PD-1 [108,109]. Li and coworkers [110] demonstrated that FLU inhibited the tumoral expression of PD-1, but not PD-L1, and the effect was concomitant to a drug-induced down-regulation of phospho-STAT3 in the tumor tissue. FLU is a potent inhibitor of the activation of STAT3 and this effect is most likely at the origin of the down-regulation of PD-1 [110,111]. It is interesting to note that FLU was found to down-regulate PD-1, but not PD-L1, whereas the related product albendazole (ABZ) has been found recently to promote ubiquitin-mediated degradation of PD-L1 in different cancer cell lines and tumor models. A clever analysis of the mechanism of action revealed that ABZ induced ubiquitination and degradation of PD-L1 by reducing the expression of protein ubiquilin 4 (UBQLN4), which is an important member of the ubiquitin-like protein family, frequently overexpressed in some cancers such as neuroblastoma and hepatocellular carcinoma [112] (Figure 4). The distinct mode of action of FLU and ABZ toward PD-1/PD-L1 calls for further studies to compare the efficacy of all members of the benzimidazole drug family. There may be useful differences to exploit and new drug combinations to design to optimize the anticancer activity of these affordable compounds.

The capacity of benzimidazole-based drugs such as FLU and ABZ to modulate the functioning of the PD-1/PD-L1 checkpoint is beneficial for their use as anticancer agents. This function could also be exploited to promote their antiparasitic effects. Interestingly, the blockade of the PD-1/PD-L1 pathway with an anti-PD-L1 antibody has been found to reduce proliferation of the parasite *Echinococcus multilocularis*, responsible for alveolar echinococcosis [113]. The PD-L1 blockade was found to modulate strongly the adaptive and innate immune response to the parasite infection, notably via an increase in activity of CD4^+^/CD8^+^ effector T cells [114]. It is of interest to determine if a similar level of regulation of the checkpoint can be achieved with a benzimidazole drug such as FLU or ABZ.

### 2.5. Other Repositioning Drug Candidates Affecting the PD-1/PD-L1 Checkpoint

**Figure 5:**
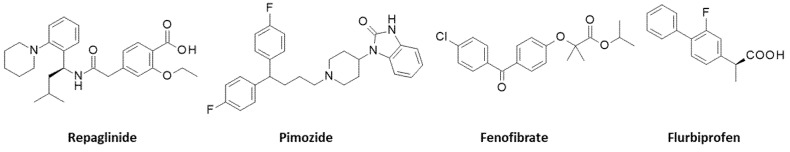
Chemical structure of four drugs impacting the expression or function of the PD-1/PD-L1 checkpoint.

The exceptional success obtained with mAbs targeting the PD-1/PD-L1 checkpoint in oncology has boosted the search for small molecules susceptible to regulating the checkpoint. Screening methods to identify compounds regulating the activity of PD-1 or PD-L1 have been designed, using either the purified proteins or engineered cell systems with direct or indirect readouts. Up- or down-regulators of PD-L1 expression have been identified from compound libraries, including natural products, kinase inhibitors, checkpoint degraders, etc. [115,116,117,118]. Several approved drugs have been characterized as well for their capacity to regulate expression and function of PD-1/PD-L1. In addition to the aforementioned drugs, we can refer to three other known drugs (Figure 5):

(i) The antidiabetic drug repaglinide is commonly used to stimulate insulin secretion from pancreatic beta cells. The drug has been shown recently to inhibit the transcription factor FOXO3 and to reduce cancer cell migration [119]. The drug has the capacity to down-regulate PD-L1 expression in glioblastoma cells [120].
(ii) The dopamine antagonist pimozide, used to treat various psychiatric diseases, has been found to display interesting antileukemic and anticancer properties. The drug can reduce activation of STAT3 and STAT5 [121,122,123]. The combination of pimozide and an anti-PD-1 agent profoundly down-regulated expression of phospho-STAT5, leading to a major antitumor response in a model of melanoma. The drug enhanced PD-1 expression, rendering the tumor more sensitive to the anti-PD-1 agent [124].
(iii) A repurposing of the lipid-lowering drug fenofibrate for the treatment of cancer has been proposed [125]. A recent work demonstrates that the drug can reprogram the immune microenvironment in a model of head and neck cancer. The drug was found to down-regulate PD-L1 expression in UMSCC47 cancer cells cultivated under hypoxic conditions (1% O_2_), probably due to a down-regulation of the upstream target hypoxia-inducible factor-1α (HIF1α) [126]. Fenofibrate has been tested in patients with multiple myeloma (NCT01965834), but not in association with an anti-PD-(L)1 mAb.
(iv) In general, β-blockers are considered not to alter the antitumor response in patients with melanoma treated with anti-PD-1 therapy [127]. It has been reported that the use of β_2_-adrenergic receptor (βAR) antagonists (β-blockers) can improve overall survival in metastatic melanoma patients who received immunotherapy [128]. The βAR is a regulator of CD8^+^ T cell frequency and functional orientation within the tumor microenvironment [129]. The repurposing of β-blockers for the treatment of triple-negative breast cancer (TNBC) has been proposed previously, based on the high expression of βAR in TNBC cell lines and the capacity of β-blockers such as propranolol to reduce their proliferation, migration, and invasion [130]. β-blockers do not directly target the PD-1/PD-L1 system, but they exert useful antiangiogenic effects. Recent clinical trials have indicated that a combination of β-blockers with anti-PD-1/PD-L1 mAbs could be useful to improve progression-free survival in patients with NSCLC [131]. The β-blocker propranolol is particularly interesting, as it has been shown not only to upregulate PD-L1 expressed on tumor-associated macrophages (TAM), but also to enhance efficacy of an anti-CTLA4 therapy in soft tissue sarcoma [132]. A recent phase 1 trial in patients with metastatic melanoma has shown that the combination of propranolol and pembrolizumab (anti-PD1) is safe (no dose-limiting toxicity observed) and preliminary signs of activity were reported (increased interferon-γ, decreased interleukin-6) [133].

Different drugs could be considered for a repositioning strategy, based on their capacity to modulate the checkpoint. This is the case, for example, with the estrogen receptor-α (ERα) antagonist fulvestrant and related selective ER down-regulators (SERDs) which have the capacity to modulate the tumor microenvironment via an action on myeloid-derived suppressor cells, tumor infiltrating lymphocytes, and other immune cell subpopulations. As such, these compounds can enhance tumor killing by CD4^+^/CD8^+^ T cells in combination with immune checkpoint inhibitors in breast cancer [134]. Other drug categories have been considered for their capacity to improve the anticancer action of immune-checkpoint inhibitors, such as renin-angiotensin system inhibitors [135], the dual endothelin receptor antagonist macitentan [136], and the antipsychotic drug trifluoperazine [137], to cite a few other examples. These effects are mostly indirect, due to the targeting of signaling pathways connected to PD-1/PD-L1, such as STAT3, STAT5, and HIF1α, and/or associated with a remodeling of the tumor microenvironment. It would be more interesting to identify drugs directly targeting the components of the checkpoint, PD-1 or PD-L1. At this level, we can refer to two drugs. On the one hand, we have previously identified the biphenyl drug flurbiprofen (Figure 5) at a potential PD-L1 binder, based on the analogy with other biphenyl compounds known to bind to PD-L1 [43]. Interestingly, this drug has been shown recently to attenuate the postoperative increase in PD-1 levels on CD8^+^ T in patients undergoing thoracoscopic surgery [138]. The use of flurbiprofen as a modulator of the PD-1/PD-L1 warrants further investigation.

**Figure 6:**
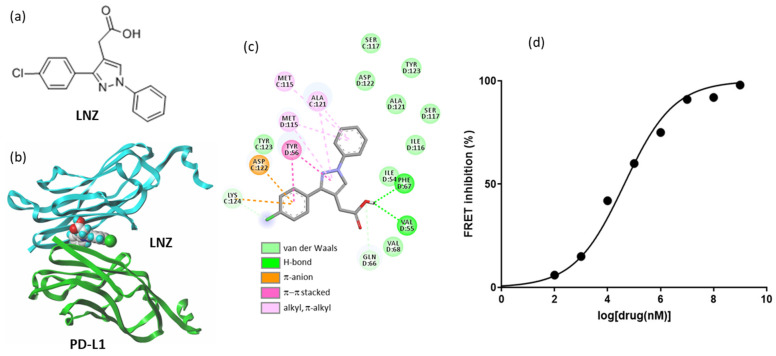
Lonazolac binding to PD-L1. (**a**) Structure of lonazolac (LNZ). (**b**) Molecular model of LNZ bound to the interface of the PD-L1 protein dimer. The two protein units (in cyan and green) sandwich the drug ligand. The binding pocket is closed by four tyrosine residues (Y56 and Y123 on each side of the cavity). (**c**) Binding map contacts for LNZ bound to PD-L1. (**d**) Dose response of LNZ on a fluorescence resonance energy transfer (FRET) assay showing that LNZ interferes with the activation of the tyrosine phosphatase SHP-2 upon its interaction with human PD-1 (from three independent experiments; IC_50_ = 43 mM).

Perhaps a more interesting drug is the old non-steroidal, anti-inflammatory drug lonazolac (LNZ), which has been largely used for the treatment of rheumatic diseases and other inflammatory pathologies. LNZ is an inhibitor of cyclooxygenase-2 and prostaglandin synthesis [139,140]. In the past, LNZ has been shown to exert anticancer activity in two syngeneic (immunocompetent) mouse models. The drug was found to reduce markedly the development of spontaneous lung metastases in C57B mice inoculated with Lewis lung carcinoma or B-16 melanoma cells [141]. We can now propose a rational basis to explain this effect because we found that LNZ can function as a modulator of PD-1 cell signaling. The interaction of LNZ with PD-L1 was evidenced using a microscale thermophoresis assay with recombinant human PD-L1 in vitro (K_D_ = 24.3 μM). A molecular model of LNZ bound to the PD-L1 dimer is shown in Figure 6. In addition, the use of a specific cell-based FRET assay revealed that LNZ interferes with the activation of the tyrosine phosphatase SHP-2 upon its interaction with human PD-1 (IC_50_ = 43 μM). The potency of LNZ as an inhibitor of PD-L1 is low compared to other small molecules specifically designed [142]. Nevertheless, it is an interesting observation that can help to design other compounds.

## 3. Discussion

Monoclonal antibodies targeting the PD-1/PD-L1 immune checkpoint have revolutionized the treatment of some forms of cancer, such as NSCLC and melanoma. For those patients, the disease outcome has been drastically improved. However, for the majority of cancer patients with an advanced-stage disease, the treatment response is limited and many of them relapse. New treatments and new drug combinations are needed [143]. The use of approved drugs able to modulate the expression and function of the immune checkpoint could represent an interesting option to promote efficacy of anti-PD-1/PD-L1 antibodies. Small molecules specifically targeting the checkpoint are being designed and developed, but the full development process is long (>10 years in most cases), excessively costly (about 1.5 b$/molecule), and at a high risk of failure [108,144]. The repositioning of an approved drug may be a more rapid and less risky alternative than the de novo development of a novel chemical entity.

**Figure 7:**
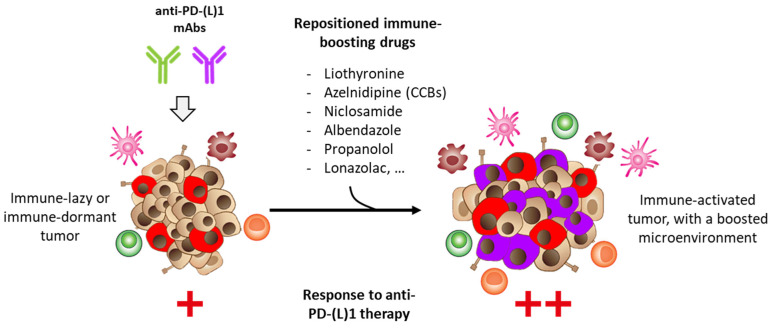
Activation of the antitumor immune system with repositioned drugs. The objective with these known drugs is to reinforce efficacy of mAbs targeting PD-1 or PD-L1, either via a direct action on the checkpoint or via indirect effectors and activation of the tumor microenvironment.

As discussed here, there are approved drugs capable of modulating the checkpoint, either by directly binding to PD-1 or PD-L1 or indirectly through the targeting of an upstream effector (e.g., STAT3, HSP90) to suppress the expression of the gene or the protein component of the checkpoint, or alternatively, to induce its degradation [145]. In all cases, the goal is to reinvigorate immunosurveillance in immune-dormant or -refractory tumors (Figure 7). Compounds such as liothyronine, azelnidipine, niclosamide, flubendazole, and a few others could be used as adjuvants to enhance the efficacy of anti-PD-1/anti-PD-L1 immunotherapies. A similar strategy has been proposed with known natural products, such as the polyphenol compound resveratrol [146,147], and with other types of enhancers of antitumor immunity [148,149,150,151]. The approach is promising. Extending the use of orally active drugs in oncology, known for their safety and efficacy to treat human diseases other than cancer, is a real bonus. The anticancer activity of these PD-1/PD-L1 immune checkpoint modulators should now be evaluated at the clinical level in combination with antibodies, as initiated recently with propranolol combined with pembrolizumab [133].

The PD-1-based repositioning approach can be further considered. Other repurposed drug candidates targeting the PD-1/PD-L1 network have been proposed, based on a bioinformatic analysis of the signaling pathway [152]. Repurposing approved drugs for cancer therapy requires a precise knowledge of the drug mechanism of action. This strategy has been largely discussed in recent years [153,154,155,156,157]. The approach can be extended to target immune checkpoints, such as PD-1/PD-L1 discussed here.

## 4. Conclusions

Low molecular weight drugs with an immunotherapeutic action against cancer are actively being searched for, such as, notably, small molecules designed to complement the activity of monoclonal antibodies directed against the PD-1/PD-L1 checkpoint. Here, we have identified a handful of well-established drugs known for their safety and efficacy in non-oncologic diseases, but are nevertheless able to modulate the activity of the checkpoint. Drugs such as liothyronine, azelnidipine, niclosamide, and flubendazole exert a specific action on the PD-L1 component, which is potentially useful to promote efficacy of anti-PD-1 mAbs. These drugs could be repositioned for use in oncology to improve the treatment of advanced cancers. Combination clinical trials with these drugs are encouraged.
